# Anti-inflammatory activity of hydrosols from Tetragonia tetragonoides in LPS-induced RAW 264.7 cells

**DOI:** 10.17179/excli2017-121

**Published:** 2017-04-18

**Authors:** Eun-Yi Ko, Su-Hyeon Cho, Kyungpil Kang, Gibeom Kim, Ji-Hyeok Lee, You-Jin Jeon, Daekyung Kim, Ginnae Ahn, Kil-Nam Kim

**Affiliations:** 1 Jeju Center, Korea Basic Science Institute (KBSI), Jeju 690-140, Republic of Korea; 2 Department of Marine Life Science, Jeju National University, Jeju 690-756, Republic of Korea; 3 BKSU Lnc. Jeju , 63243, Republic of Korea; 4 Department of Marine Bio-food science, College of Fisheries and Ocean Sciences, Chonnam National University, 500-749, Republic of Korea; 5 Chuncheon Center, Korea Basic Science Institute (KBSI), Chuncheon 24341, Republic of Korea

**Keywords:** Tetragonia tetragonoides, hydrosols, anti-inflammatory, NF-kappaB, MAPKs

## Abstract

The present study was performed to investigate the anti-inflammatory activity of *Tetragonia tetragonoides* hydrosols (TTH) and its underlying mechanism in lipopolysaccharide (LPS)-induced RAW 264.7 cells. Gas chromatography (GC) coupled with mass spectrometry and retention index calculations showed that TTH were mainly composed of tetratetracontane (29.5 %), nonacosane (27.6 %), and oleamide (17.1 %). TTH significantly decreased the production of nitric oxide (NO), prostaglandin E2 (PGE_2_), interleukin (IL)-6, and IL-1β in LPS-stimulated RAW 264.7 cells. Consistent with these observations, TTH treatment decreased the protein expression levels of inducible NO synthase (iNOS) and cyclooxygenase-2 (COX-2). The molecular mechanism of its anti-inflammatory activity was found to be associated with inhibition of nuclear factor-kappa B (NF-κB) phosphorylation and nuclear translocation of NF-κB 65. Furthermore, TTH markedly suppressed the LPS-induced phosphorylation of mitogen-activated protein kinases (MAPKs). Taken together, these data indicate that TTH exerts an anti-inflammatory activity by inhibiting the NF-κB and MAPK signaling pathways in LPS-stimulated RAW 264.7 cells.

## Introduction

Inflammation is a process involving cellular events that occur in response to infection and tissue injury and is required for the maintenance of good health in response to bacterial and viral infections. However, excessive or prolonged inflammation can be harmful and may contribute to the pathogenesis of a variety of diseases, including rheumatoid arthritis, atherosclerosis, cancer, periodontitis, and chronic hepatitis (Bosca et al., 2005[8]; Medzhitov, 2008[20]). It has been reported that the excessive production of pro-inflammatory mediators and cytokines, such as nitric oxide (NO), prostaglandin E2 (PGE_2_), tumor necrosis factor-α (TNF-α), interleukin (IL)-1, and IL-6 plays an important role in the development of these inflammatory diseases (Dinarello, 1997[10]; Wei et al., 2015[30]). Thus, reduction in the production of these inflammatory mediators and cytokines is an important target in the treatment of inflammatory diseases.

Hydrosols are by-products produced during water or steam distillation of plant material. Since they contain trace amounts of essential oils as well as hydrophilic dissolved compounds, hydrosols are commonly used for aroma therapy and cosmetic purposes (Inouye et al., 2009[13]; Lante and Tinello, 2015[18]). Hydrosols have great capability to meet the demands of the food and cosmetic industries, because they are not only easy and inexpensive to produce but are also non-toxic. Moreover, they have been reported to exhibit several biological activities such as antimicrobial (Inouye et al., 2009[13]; Kunicka-Styczyńska et al., 2015[17]), antifungal (Ozturk, 2015[24]), anti-tyrosinase (Lante and Tinello, 2015[18]), and antioxidant (Prusinowska et al., 2016[28]). However, only few studies have investigated the anti-inflammatory effect of hydrosols (Boukhatem et al., 2014[9]). 

*Tetragonia tetragonoides* is an annual herb that widely grows as littoral and estuarine species in Korea, Japan, southeast China, New Zealand, Kermadec Islands, New Caledonia, Hawaii, and other Pacific Islands (Aoki et al., 1982[4]; Hara et al., 2008[12]; Kato et al., 1985[14]). *T. tetragonioides* can be ingested as a food crop, such as a salad or herb, and has been used as a medicinal plant for the treatment of gastric ulcer, gastritis, and gastroenteritis (Aoki et al., 1982[4]; Kato et al., 1985[14]). However, the anti-inflammatory effect of hydrosols extracted from *T. tetragonoides* (TTH) has not been reported. In this study, the anti-inflammatory effect of TTH was investigated using lipopolysaccharide (LPS)-induced RAW 264.7 cells.

## Materials and Methods

### Reagents

Dimethyl sulfoxide (DMSO), RIPA buffer, and phosphate-buffered saline (PBS) were purchased from Sigma-Aldrich (St. Louis, MO, USA). Dulbecco's modified Eagle's medium (DMEM) and fetal bovine serum (FBS) were obtained from Invitrogen-Gibco (Grand Island, NY, USA). ELISA kits for PGE_2_, IL-1β, and IL-6 were purchased from R&D Systems, Inc. (St. Louis, MO, USA) and BD Biosciences (San Diego, CA, USA). Anti-phosphorylated IκB-α (anti-p-IκB-α), anti-NF-κB, anti-JNK, anti-phosphorylated JNK (anti-p-JNK), anti-ERK1/2, anti-phosphorylated ERK1/2 (anti-p-ERK1/2), anti-p38, and anti-phosphorylated p38 (anti-p-p38) mouse or rabbit antibodies were purchased from Cell Signaling Technology (Beverly, MA, USA). All other reagents were purchased from Sigma-Aldrich Chemical Co. (St. Louis, MO, USA).

### Extraction of hydrosols from Tetragonia tetragonoides

*T. tetragonoides* were collected from Jeju Island in the Republic of Korea. The samples were washed with water to remove salt and sand, rinsed carefully in fresh water, and then dried at room temperature for 1 week. Dried *T. tetragonoides* (2 kg) were soaked in water (80 L) and then distilled at 121 °C for 4 h using a high-speed, low-temperature vacuum extractor (DM-3000, Daehan median co., LTD, Seoul, Korea). The primary oil decanted from distillate (hydrosols) was filtered to remove particles and stored in a sealed vial at 4 °C until further testing.

### GC-MS analysis

Composition analysis of volatile oil was performed by gas chromatography-mass spectrometry (GC-MS) using Shimadzu GCMS-TQ8040 (Shimadzu Corporation, Japan) on a Rxi-5MS capillary column (30 m × 0.25 mm id, film thickness 0.25 μm; Shimadzu, Japan). The carrier gas was helium (flow rate of 1.36 ml/min). GC-MS was carried out using the following temperature program: initial temperature was set at 180 °C and held for 3 min, followed by 6 °C/min ramp to 240 °C and hold for 3 min, followed by 3 °C/min ramp to 275 °C and hold for 3 min, followed by 5 °C/min ramp to 300 °C and hold for 3 min, followed by 5 °C/min ramp to a final temperature of 330 °C and hold for 5 min. The injection temperature was set at 290 °C and the injection volume was 1 μl (splitless mode). Detector parameters used for GC-MS analyses were as follows: interface temperature, 320 °C; ion source temperature, 300 °C; mass spectrometry was performed using Q3 scan with an m/z 45-500 scanning range. Chromatograms and mass spectra were evaluated using the GCMS solution software (Shimadzu Corporation, Japan).

### Cell culture

Raw 264.7 murine macrophage cells were purchased from the Korean Cell Line Bank (KCLB; Seoul, Korea). The cells were cultured in DMEM supplemented with antibiotics (100 U/ml penicillin and 100 μg/ml streptomycin) and 10 % FBS at 37 °C in a 5 % CO_2_ atmosphere.

### Cell viability assay

RAW 264.7 cells (2 × 10^5^ Cells/well) were plated in 96-well plates and incubated overnight, and subsequently treated with LPS (1 μg/ml) in the absence or presence of various concentrations (0.5 %-5 %) of TTH at 37 °C for 24 h. After incubation, MTT solution (5 mg/ml) was added to each well and plates were incubated for 4 h in a CO_2_ incubator at 37 °C. Subsequently, supernatant was removed and DMSO was added to dissolve formazan crystals. The absorbance was measured at 540 nm.

### Measurement of NO production 

RAW 264.7 cells (2 × 10^5 ^Cells/well) were plated in 24-well plates and subsequently treated with LPS (1 μg/ml) in the absence or presence of various concentrations (0.5 %-5 %) of TTH at 37 °C for 24 h. Culture supernatants were mixed with Griess reagent (1:1 mixture of 1 % sulfanilamide and 0.1 % naphthylethylenediamine dihydrochloride in 2.5 % phosphoric acid) and incubated at room temperature for 10 min. Subsequently, the NO concentration in supernatant was determined by measuring absorbance at 540 nm.

### Measurement of cytokines (IL-1β and IL-6) and PGE_2_ by ELISA

RAW 264.7 cells (2 × 10^5^ Cells/well) were plate in 24-well plates and subsequently treated with LPS (1 μg/ml) in the absence or presence of various concentrations (0.5 %-5 %) of TTH at 37 °C for 24 h. Culture supernatants were collected for determination of IL-1β, IL-6, and PGE_2_ concentrations by ELISA according to the manufacturer's instructions.

### Western blot analysis

RAW 264.7 macrophages were cultured at 2 × 10^5^ cells per 35 mm dish and incubated at 37 °C for 24 h. The cells were then treated with different concentrations of TTH and incubated for 24 h. The cells were lysed using RIPA lysis buffer. Protein concentrations were determined using a Bio-Rad protein assay kit; bovine serum albumin (BSA) was used as the calibration standard. Cell lysates were electrophoresed on SDS polyacrylamide gels (8-12 %) and the separated proteins were transferred to PVDF membranes. The membranes were incubated in the blocking solution (Tris-buffer /Tween 20, TBST) containing 3 % BSA (w/v) for 2 h under gentle shaking at room temperature. Subsequently, the membranes were incubated with primary antibodies (iNOS, COX-2, p-p65, p-p105, p-IκB-α, p65, p105, IκB, p-ERK, ERK, p-JNK and JNK diluted 1:1000 in 3 % BSA in TBST) for 24 h at 4 °C. After incubation, the membranes were washed three times with TBST buffer at room temperature, and then incubated with secondary antibodies (1:3000) in 3 % BSA in TBST for 2 h at room temperature. Signals were developed using ECL Western blotting detection kit and visualized on a Bio-Rad ChemiDoc system. The density of the band was quantified using ImageJ software (National Institutes of Health).

### Confocal microscopy analysis

Cells were fixed in 4 % formaldehyde for 15 min at room temperature and permeated with methanol at −20 °C for 10 min. Cells were then blocked for 1 h with 1 % BSA in PBS and permeabilized by incubation with 0.4 % Triton X-100 for 30 min. Subsequently, cells were washed with PBS and incubated with anti-p65 primary antibody (1:100) at 4 °C overnight. After washing with PBS, cells were incubated with a secondary antibody (1:100) at 4 °C for 1.5 h. Cells were then washed with PBS and incubated with Alexa Fluor 488 goat anti-rabbit antibody (1:800) for 1.5 h at RT in the dark. After washing with PBS, slides were mounted and counter-stained using Vectashield mounting medium containing DAPI (Vector Laboratories, Burlingame, CA, USA). Slides were visualized under a laser scanning microscope (LSM 700; ZEISS, Jena, Germany).

### Statistical analysis

All data are expressed as means ± S.D. Significant differences among the groups were determined using the unpaired Student's *t*-test. A value of *p < 0.05 was considered to be statistically significant.

## Results

### Chemical composition of TTH

The chemical profile, percentage content, and retention indices of the constituents of TTH are summarized in Table 1(Tab. 1). The following six compounds were identified in TTH, representing 86.4 % of hydrosols: tetratetracontane (29.5 %, nonacosane (27.6 %), oleamide (17.1 %), octacosane (7.6 %), 2,6,10,15-tetramethyl-heptadecane (2.6 %), and 2-methyleicosane (2.0 %).

### Effect of TTH on the production of NO and PGE_2_ in LPS-stimulated RAW 264.7 cells

**Figure 1:**
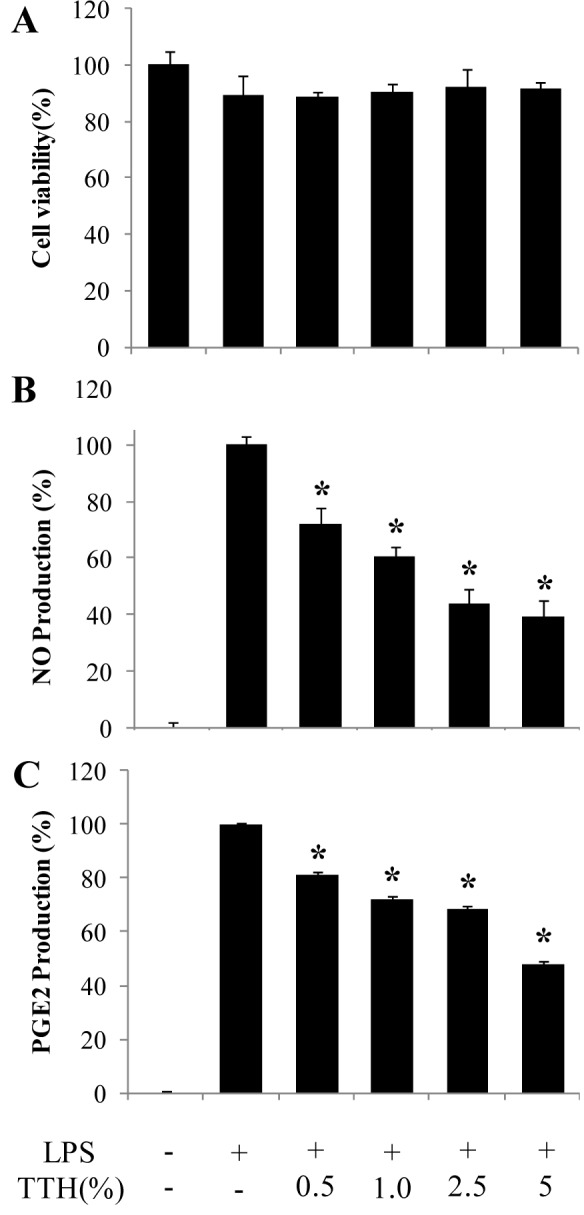
Effects of TTH on (A) cell viability and (B) NO and (C) PGE_2_ production in LPS-induced RAW 264.7 cells. Cells were pretreated for 1 h with different concentrations (0.5 %, 1 %, 2.5 % and 5 %) of TTH and then LPS (1 μg/ml) was added and incubated for 24 h. Cell viability was determined using the MTT assay. Values are expressed as means ± S.D. of triplicate experiments. *P < 0.05 indicate significant differences from the LPS-stimulated group.

We first treated the cells with TTH and measured the cytotoxicity using MTT assay to determine the non-cytotoxic concentration (Figure 1A(Fig. 1)). In all subsequent experiments, TTH was used at concentrations ranging from 0.5 % to 5 %. To determine whether TTH inhibited the production of LPS-induced NO and PGE_2_, which play a central role in the inflammatory response, RAW 264.7 cells were pretreated with TTH for 1 h and then stimulated with LPS. Compared to that in unstimulated cells, NO and PGE_2_ production was markedly induced in LPS-stimulated cells. TTH significantly inhibited the production of LPS-induced NO and PGE_2_ in a concentration-dependent manner (Figure 1(Fig. 1)). 

### Effects of TTH on iNOS and COX-2 protein expression in LPS-stimulated RAW 264.7 cells

**Figure 2:**
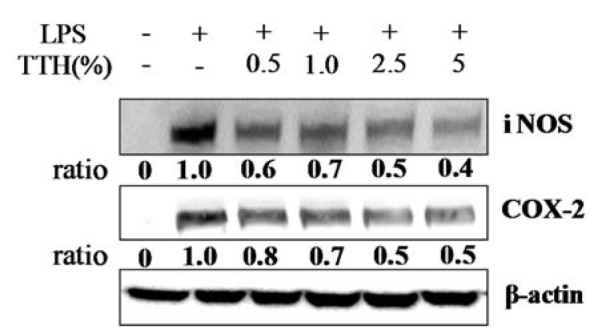
Effects of TTH on LPS-induced iNOS and COX-2 protein expressions in RAW 264.7 cells. Cells were treated for 1 h with different concentrations (0.5 %, 1 %, 2.5 % and 5 %) of TTH, LPS (1 μg/ml) was then added and cells were incubated for 24 h. iNOS and COX-2 protein level were determined via Western blotting. Density ratio of a TTH treated-group over LPS only treated-group was measured by densitometer.

RAW 264.7 cells were treated with TTH to study the expression of inflammation-related proteins induced by LPS treatment. The LPS-stimulated RAW 264.7 cells showed significant iNOS and COX-2 protein expression. However, treatment with TTH dose-dependently inhibited iNOS and COX-2 protein expression in LPS-stimulated RAW 264.7 cells (Figure 2(Fig. 2)). 

### Effect of TTH on the production of pro-inflammatory cytokines in LPS-stimulated RAW 264.7 cells

**Figure 3:**
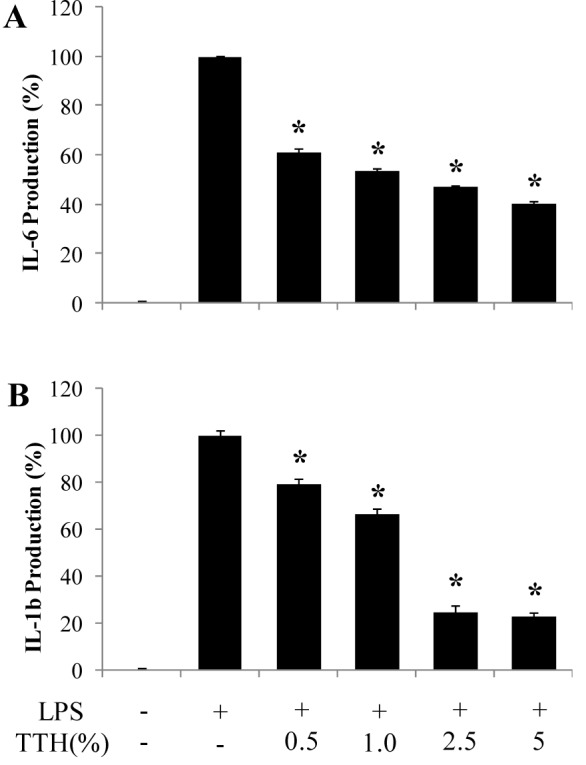
Effect of TTH on the pro-inflammatory cytokine production in LPS-induced RAW 264.7 cells. The production of (A) IL-6 and (B) IL-1β were assayed in the culture medium of cells stimulated with LPS (1 μg/ml) for 24 h in the presence of TTH (0.5 %, 1 %, 2.5 % and 5 %)). Supernatants were collected, and the IL-1β, IL-6, and TNF-α concentration in the supernatants were determined by ELISA. Values are expressed as means ± S.D. of triplicate experiments. *P < 0.05.

To further investigate the anti-inflammatory effect of TTH on LPS-stimulated macrophages, the production of pro-inflammatory cytokines was evaluated by ELISA. The treatment of RAW 264.7 cells with LPS significantly increased IL-6 and IL-1β production compared to that in the untreated (control) cells. However, TTH treatment significantly and dose-dependently decreased the levels of IL-1β and IL-6 compared to that reported with the LPS treatment (Figure 3(Fig. 3)). 

### Effect of TTH on NF-κB and MAPK activation in LPS-stimulated RAW 264.7 cells

**Figure 4:**
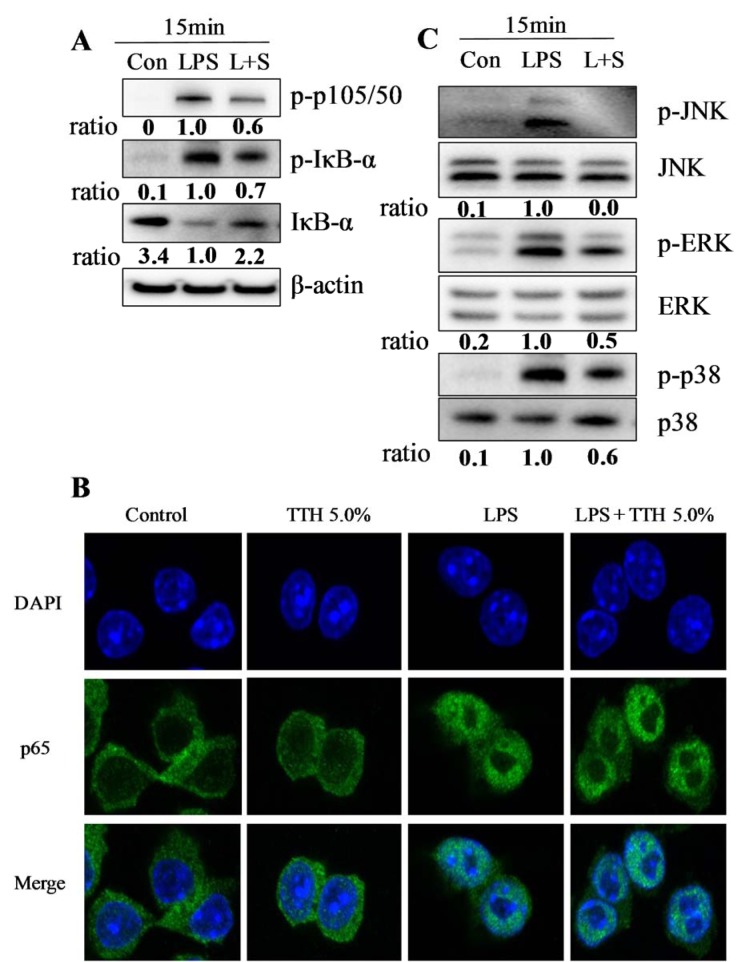
Inhibitory effect of TTH on phosphorylation of MAPKs and NF-κB activation in LPS-induced RAW 264.7 cells. Cells were treated for 15 min with LPS (1 μg/ml) alone or with LPS (1 μg/ml) coupled with 5 % TTH. The expression levels of (A) NF-κB and (C) MAPKs protein were determined via Western blotting. Density ratio of a TTH treated-group over LPS only treated-group was measured by densitometer. (B) The p65 protein localization in cells was determined with an anti-p65 antibody and Alexa Fluor 488 goat anti-rabbit antibody by laser confocal scanning microscopy.

Nuclear translocation of NF-κB is directly linked to IκB-α degradation and phosphorylation and is initiated by the phosphorylation, ubiquitination, and proteolytic degradation of IκB-α (Ghosh and Karin, 2002[11]). Therefore, the levels of phosphorylated IκB-α were examined by Western blot analysis. As shown in Figure 4A(Fig. 4), unstimuated RAW 264.7 cells did not show detectable form of phosphorylated IκB-α, whereas addition of LPS resulted in phosphorylation and degradation of IκB-α. However, pre-treatment with TTH markedly inhibited the LPS-induced phosphorylation and degradation of IκB-α. For further confirmation, the nuclear translocation of NF-κB/p65 was visualized under a laser confocal microscope. The LPS-stimulated RAW 264.7 cells showed a dramatic increase in the translocation of NF-κB into the nucleus. The LPS-stimulated NF-κB nuclear translocation was markedly suppressed after TTH pre-treatment (Figure 4B(Fig. 4)). These results suggest that TTH inhibits IκB-α degradation and prevents NF-κB translocation into the nucleus.

MAPKs are associated with the production of inflammatory mediators in marcrophages (Uto et al., 2005[29]). Therefore, western blot analysis was performed to determine whether TTH mediates its effect through the MAPK pathway. The LPS-induced phosphorylation of p38, ERK1/2, and JNK was evaluated in RAW 264.7 cells. As shown in Figure 4C(Fig. 4), LPS stimulation significantly increased the phosphorylation of MAPK, whereas TTH treatment markedly inhibited the expression of phosphorylated MAPK in LPS-stimulated cells (Figure 4C(Fig. 4)).

## Discussion

The present study revealed that TTH shows anti-inflammatory activity. To evaluate the anti-inflammatory activity, cells were pretreated with TTH and then stimulated with LPS. In macrophages, LPS, a well-known endotoxin, induces the production of inflammatory cytokines such as IL-6 and IL-1β, and inflammatory mediators such as NO and PGE_2_, which are synthesized by iNOS and COX-2, respectively (Becker et al., 2005[6]; Kim et al., 2010[15]; Posadas et al., 2000[27]). The overproduction of NO and PGE_2_ has been reported to contribute to the pathogenesis of inflammatory diseases, including rheumatoid arthritis, atherosclerosis, chronic hepatitis, pulmonary fibrosis, and inflammatory brain diseases (Baraf, 2007[5]; Bosca et al., 2005[8]; Minghetti, 2004[21]; Nathan, 1992[22]). Pro-inflammatory cytokines are predominantly produced by activated macrophages and they promote inflammatory reactions. It is well known that certain pro-inflammatory cytokines, such as IL-1β, IL-6, and TNF-α, are involved in the pathogenesis of pain (Andreakos et al., 2004[2]; Yan and Hansson, 2007[31]; Zhang and An, 2007[32]). Therefore, reduction of NO, PGE_2_, and pro-inflammatory cytokine levels may be an effective strategy for inhibiting inflammation. In this study, TTH treatment reduced NO and PGE_2_ production by regulating iNOS and COX-2 protein expression. TTH also reduced the LPS-stimulated production of IL-1β and IL-6, indicating that it has an anti-inflammatory activity in LPS-stimulated RAW 264.7 macrophages. These activities may be attributed to the individual or synergistic action of tetratetracontane, nonacosane, oleamide, octacosane, 2,6,10,15-tetramethyl-heptadecane, and 2-methyleicosane components found in TTH. Nevertheless, anti-inflammatory activity of TTH could be mainly attributed to the presence of oleamide. Oleamide has been reported to have significant anti-inflammatory activity (Ano et al., 2015[3]). For example, Oh et al. (2010[23]) reported that oleamide treatment significantly inhibited the LPS-induced production of pro-inflammatory mediators and activation of NF-κB in BV2 murine microglial cells. 

NF-κB regulates the expression of various genes that play an essential role in cell proliferation, tumorigenesis, and inflammation (Pikarsky et al., 2004[26]). It is known that NF-κB plays a central role in the LPS-induced transcriptional regulation of inflammatory genes (Kim et al., 2013[16]; Pikarsky et al., 2004[26]). LPS stimulation causes rapid phosphorylation and degradation of IκB-α. This subsequently results in an increase in p65/p50 heterodimer that translocates into the nucleus (Li and Verma, 2002[19]; Pahl, 1999[25]). After translocation, it binds to its target sites and induces the transcription of pro-inflammatory mediators and pro-cytokines (Li and Verma, 2002[19]; Pahl, 1999[25]). We found that TTH suppressed the LPS-induced IκB-α phosphorylation and degradation, and translocation of NF-κB p65. These results indicate that TTH exerts inhibitory effects on the production of inflammatory mediators and cytokines by regulating the NF-κB signaling pathway. 

MAPKs including p38, extracellular signal-regulated kinase (ERK), and c-Jun N-terminal kinase (JNK) have important functions such as regulation of cell differentiation, cell growth, and cellular responses to cytokines in the immune system. MAPKs regulate inflammatory and immune responses and the MAPK signaling pathways are involved in LPS-induced COX-2 and iNOS expression in macrophages (Ajizian et al., 1999[1]; Uto et al., 2005[29]). p38 is involved in regulating the expression of iNOS and TNF-α genes in macrophages, whereas ERK and JNK are involved in regulation of pro-inflammatory cytokines and iNOS (Bhat et al., 1998[7]; Uto et al., 2005[29]). Results of the present study showed that TTH inhibited the LPS-induced phosphorylation of all three MAPKs. This indicates that the TTH-mediated inhibition of LPS-induced pro-inflammatory mediators is partly caused by inhibition of the MAPK signaling pathways. 

In conclusion, the present study showed that TTH treatment inhibited the LPS-induced NO and PGE_2_ production in RAW 264.7 cells by suppressing iNOS and COX-2 expression, respectively. TTH also inhibited the LPS-induced production of IL-1β and IL-6 in RAW 264.7 cells. These effects could have been exerted by the inhibition of NF-κB activation and phosphorylation of the MAPK pathways. These findings provide evidence that TTH exhibits potential anti-inflammatory activities.

## Notes

Eun-Yi Ko and Su-Hyeon Cho contributed equally to this work.

## Acknowledgements

This work was supported by a Korea Basic Science Institute (C37965) and (C37260).

## Conflict of interest

The authors declare no conflict of interest.

## Figures and Tables

**Table 1:**
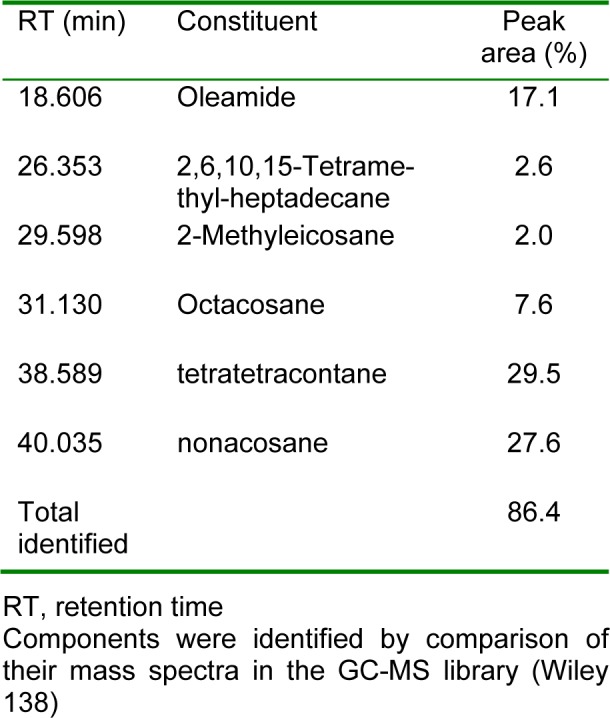
Chemical composition of the TTH
